# PHYSICAL SYMPTOM TRAJECTORIES OF OLDER ADULTS DURING THE COVID-19 PANDEMIC

**DOI:** 10.1093/geroni/igac059.3014

**Published:** 2022-12-20

**Authors:** Dylan Lee, Soyoung Choun, Hye Soo Lee, Maria Kurth, Carolyn Aldwin

**Affiliations:** Touro University Nevada, Henderson, Nevada, United States; Oregon State University, Corvallis, Oregon, United States; Oregon State University, Corvallis, Oregon, United States; Oregon State University, Corvallis, Oregon, United States; Oregon State University, Corvallis, Oregon, United States

## Abstract

Older adults experience increased risk for morbidity and mortality during the COVID-19 pandemic (CDC, 2021). Social distancing and lockdown to prevent contagion may affected physical and mental health. We examined the fifteen physical symptom trajectories of older adults during the early months of the COVID-19 pandemic. We also examined age, gender, and marital status differences in each physical symptom trajectory. The sample consisted of 247 older adults (Mage = 71.1, SD = 7.3, 88.7% White, 73% women, 73.4% married), who participated in eight weekly longitudinal online surveys from April 28 to June 23, 2020. Random-effects logistic regression analysis controlling for age, gender, marital status, and depressive symptoms showed that the nine physical symptoms (headache, constipation/diarrhea, muscle soreness, shortness of breath, tightness of chest, backache, heart pounding, congestion, and sore throat) significantly decreased in the first few weeks, but then six symptoms (constipation/diarrhea, shortness of breath, tightness of chest, heart pounding, congestion, and sore throat) increased in later weeks of the study period. Middle-aged participants reported higher number of headache, tightness of chest, heart pounding, and nausea/upset stomach than older participants. Women experienced more constipation/diarrhea, trembling/shaking, and sore throat than men. Those who were not married responded with higher number of shortness of breath, backache and poor appetite compared to married participants. Higher depressive symptoms were significantly related to each physical symptom. The results suggest that healthcare providers should evaluate physical symptoms focusing on the patients who are at greater risk of poor health during the pandemic.

